# Real-time positioning in logging: Effects of forest stand characteristics, topography, and line-of-sight obstructions on GNSS-RF transponder accuracy and radio signal propagation

**DOI:** 10.1371/journal.pone.0191017

**Published:** 2018-01-11

**Authors:** Eloise G. Zimbelman, Robert F. Keefe

**Affiliations:** Department of Forest, Rangeland and Fire Sciences, University of Idaho, Moscow, ID, United States of America; The University of Sydney, AUSTRALIA

## Abstract

Real-time positioning on mobile devices using global navigation satellite system (GNSS) technology paired with radio frequency (RF) transmission (GNSS-RF) may help to improve safety on logging operations by increasing situational awareness. However, GNSS positional accuracy for ground workers in motion may be reduced by multipath error, satellite signal obstruction, or other factors. Radio propagation of GNSS locations may also be impacted due to line-of-sight (LOS) obstruction in remote, forested areas. The objective of this study was to characterize the effects of forest stand characteristics, topography, and other LOS obstructions on the GNSS accuracy and radio signal propagation quality of multiple Raveon Atlas PT GNSS-RF transponders functioning as a network in a range of forest conditions. Because most previous research with GNSS in forestry has focused on stationary units, we chose to analyze units in motion by evaluating the time-to-signal accuracy of geofence crossings in 21 randomly-selected stands on the University of Idaho Experimental Forest. Specifically, we studied the effects of forest stand characteristics, topography, and LOS obstructions on (1) the odds of missed GNSS-RF signals, (2) the root mean squared error (RMSE) of Atlas PTs, and (3) the time-to-signal accuracy of safety geofence crossings in forested environments. Mixed-effects models used to analyze the data showed that stand characteristics, topography, and obstructions in the LOS affected the odds of missed radio signals while stand variables alone affected RMSE. Both stand characteristics and topography affected the accuracy of geofence alerts.

## Introduction

Since the Department of Defense launched its first Navigation System with Timing and Ranging (NAVSTAR) satellite in 1978, global positioning system (GPS) technology has become an integral component of national defense, homeland security, civilian life, and scientific research [1]. Global navigation satellite system (GNSS) positioning is a more general term that encompasses all global satellite-based positioning systems such as GPS, GLONASS, Galileo, and BeiDou. Initial research evaluating the practicality of GNSS technology in forested landscapes indicated the potential use of GNSS for a range of operational and research uses in natural resources [2]. Early assessments of GNSS for forestry found it could be used to locate forest inventory plots [3,4], quickly determine timber harvest unit boundaries [4], locate forest roads [4], and track wheeled skidders [5]. Various other studies tracked harvest equipment with GNSS and used this information to assess site disturbance and to calculate productivity from time-study data [6,7]. More recent studies related to forest operations have analyzed the potential for GNSS data to quantify production efficiency [8], track log trucks [9], improve operational monitoring [10], and increase efficiency and calibrate remotely-sensed inventory data using GNSS-derived harvester head positions [11].

The accuracy of GNSS coordinate readings is dependent upon the number and geometry of satellites visible to a GNSS unit at any point in time. Positional dilution of precision (PDOP) is an index of the influence of satellite geometry on GNSS measurements [12]. In general, a lower PDOP value indicates an arrangement of satellites providing higher measurement reliability and values less than 2 are desirable [12]. PDOP values can be calculated for user-defined locations using GNSS mission planning software [13]. Another factor that could potentially affect GNSS accuracy in forested conditions is aspect. The Wide Area Augmentation System (WAAS) provides real-time GNSS data correction [14]. GNSS receivers need a clear view of a geostationary communications satellite (GEO) in order to receive WAAS correction signals [14]. GNSS receivers in the northern United States usually need an unobstructed view to the south of less than 20 degrees to receive WAAS signals because GEO satellites are low on the horizon [14]. This leads to a potential for increased error on slopes without a clear view to the south [14]. Few studies have evaluated the effect of aspect on GNSS performance, and while two studies found higher GNSS fix rates and lower location error on south aspects, these differences were not statistically significant [15,16].

GNSS use in forestry is often affected by error associated with satellite signal obstruction by the canopy or other solid objects and the reflection or diffraction of satellite signals from nearby objects or surfaces, an error known as multipathing [13,17]. Previous studies have shown that forest stand structural characteristics and terrain affect GNSS accuracy [5,18–23]. Holden et al. [21] developed a method to model GNSS precision using three canopy descriptor variables (percentage of sky obstruction, maximum canopy hole radius, and fragmentation of sky view). Lewis et al. [22] modeled the proportion of 3D GNSS fixes, PDOP, and location error using the percent canopy cover and satellite view (to represent terrain obstruction). Newer studies have taken advantage of the correlation between GNSS signal strength and forest stand characteristics by evaluating the potential to predict and map forest parameters using GNSS signals [24,25]. GNSS receiver type (survey-, mapping-, or recreation-grade) also affects the accuracy of position measurements [5,26–30]. Survey-grade receivers are capable of sub-centimeter accuracy in the open and sub-meter accuracy under mature forest conditions [30,31]. At the other end of the spectrum, recreation-grade units are the least expensive and have accuracies ranging from 2–5 m in the open [13,32–34] to 3.8–12 m in mature forests [13,30,32–34].

Advances in positioning technology for remote environments have emerged simultaneously for several uses, ranging from recreation to public safety and defense. These devices link GNSS positional information with radio frequency (RF) transmission of location coordinates (GNSS-RF) to form ad-hoc networks in which the locations of all units can be monitored on mobile phones or tablets. Like traditional GNSS devices, each GNSS-RF transponder determines its coordinates using one or more satellite-based positioning systems. However, the RF transmission is a second component that allows those coordinates to be sent to other, nearby GNSS-RF units at user-defined intervals. GNSS-RF transponders include consumer-grade units for recreational use like the Garmin Rino and Garmin Alpha 100, devices such as the Raveon Atlas PT marketed for public safety, a variety of military-grade GNSS radios designed for defense applications, and consumer-grade mobile-based solutions from goTenna and Beartooth that turn smartphones into two-way radios for voice or text communication in areas without cellular service. While traditional GNSS devices allow users to see their own positions, GNSS-RF devices enable real-time positioning through location sharing among individuals and equipment in remote locations [8,23,35–37]. Thus, either the device’s native screen or an attached tablet can display the location of other devices in the network moving in real-time.

Many GNSS-RF transponders support geofencing, in which a virtual boundary is defined around a user-defined geographic zone. Geofences are either circular or polygonal in shape, can vary widely in size depending on intended application, and can be stationary or mobile. Alert notifications are triggered as tracked mobile objects cross into or out of the geofence, and this functionality may be useful for a range of operational forestry applications such as detecting the amount of time workers spend near cable logging hazards [23], signaling when log skidders or log trucks cross harvest unit boundaries [36], and delineating tree falling hazard zones around manual fallers [37]. GNSS-RF real-time positioning and geofences have the potential to improve communication and situational awareness on logging operations, in wildland firefighting, transportation, and recreation. In this study, our focus was on logging safety, as logging is consistently ranked as one of the most hazardous occupations in the United States [38], with the highest fatal work injury rate of civilian occupations in 2015 [39]. By increasing situational awareness, the active display of real-time positioning logistics may be able to reduce hazards posed by the frequent interactions among ground workers, heavy equipment, and irregular terrain that are common on active logging operations.

GNSS-RF and related technologies pose new challenges for quantifying positional accuracy because positional error is associated with both the accuracy of GNSS locations and successful propagation of radio signals between devices. In other words, the accuracy of GNSS-RF position sharing depends not only on factors that influence GNSS accuracy as described above, but also on factors that affect radio signal propagation and attenuation. Radio signals experience diffraction, scattering and reflection as they travel through vegetation [40–42]. Leaf state and vegetation depth and density influence radio signal attenuation [42–45]. Attenuation can also be affected by wind [42,43,46], humidity [47,48], rain [46], and terrain [49,50]. It is unclear whether the same factors affect both the GNSS and RF components of emerging technologies, or if different forest stand characteristics and topographic factors affect one or the other.

The purpose of this study was to characterize the factors affecting real-time positioning on irregular, forested terrain through analysis of the effects of forest stand characteristics, topography and other line-of-sight (LOS) obstructions on the GNSS accuracy and radio signal propagation quality of multiple Raveon Atlas PT GNSS-RF transponders (Raveon Technologies, Vista, California, USA) functioning as a network. Until recently, most previous research evaluating GNSS for forestry applications has focused on stationary units. Recent studies characterizing GNSS units in motion include work by Kaartinen et al. [11] and Liu et al. [24], while others [8,23,36,37] have quantified mobile GNSS units paired with RF-based transmission. To further our understanding of location sharing among mobile GNSS units, we evaluated the time-to-signal accuracy of geofence crossing alerts in 21 randomly-selected stands on the University of Idaho Experimental Forest, under a wide spectrum of stand and topographic conditions. Time-to-signal accuracy refers to the difference between when a person or object crosses a geofence and when the alert is generated and shared with other, nearby GNSS-RF devices. Specifically, we tested three hypotheses. Our first null hypothesis was that neither forest stand characteristics, topography, nor the presence of obstructions in the line-of-sight affected the probability of successful radio signal propagation between GNSS-RF units. We tested this by analyzing the odds of missed radio signals within each stand using mixed-effects logistic regression. Our second null hypothesis was that neither stand characteristics, topography, nor physical obstructions affected the stationary positional accuracy of GNSS-RF units. To test this, we used linear mixed-effects models to determine which factors most affected root mean squared error (RMSE) of the PT. Our third null hypothesis was that neither stand characteristics, topography, nor physical obstructions affected the time-to-signal accuracy of geofence crossings. We tested this by using linear mixed-effects models to determine which factors most affected geofence intersection alert delay.

## Materials and methods

### Field experiment

Five Raveon Atlas PT GNSS-RF transponders collected positional data during the field experiment. As GNSS-RF units, the PTs receive their coordinates and then transmit that information to other PTs using radio frequency. The units can be attached to tablets or computers, which allows ground workers and equipment operators on logging operations to see all other positioning devices in real-time. PTs receive their coordinates from NAVSTAR GPS satellites only and have a specified 24-hour static accuracy of < 2.5 m for 50% of measurements and of < 5 m for 90% of measurements [51]. Depending on terrain, the devices can communicate up to 48 km away and position updates can be transmitted as frequently as one signal per second [51]. PTs can be used with Raveon RavTrack software, which has several options for geofencing, including different notification options.

**Fig 1:**
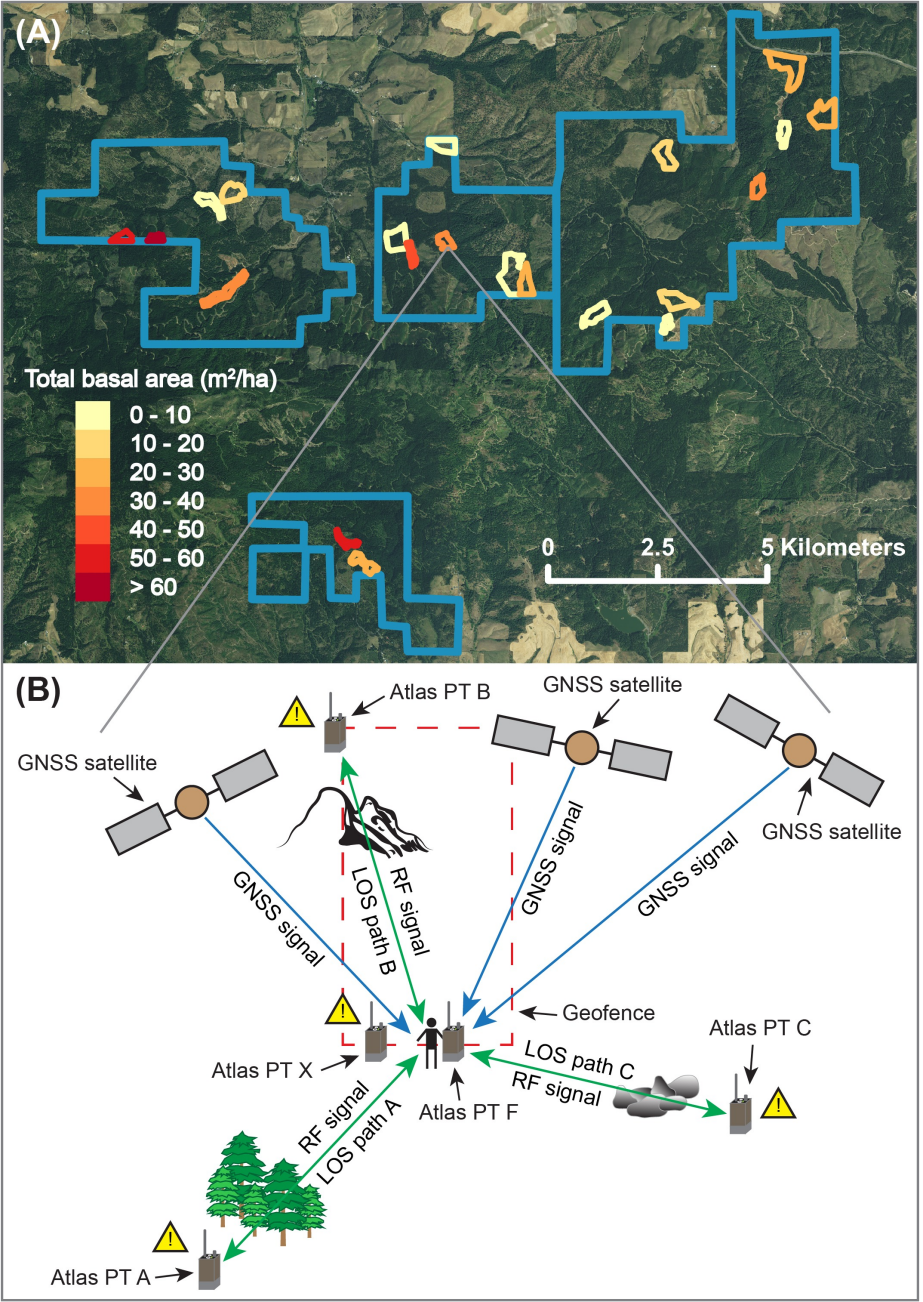
Map of the stand locations on the UIEF and illustration of the experimental setup. (A) The 21 stands are delineated according to total basal area (m^2^/ha) and the UIEF unit boundaries are shown in blue. Background map is 1-m NAIP imagery. (B) Illustration of global navigation satellite system (GNSS) technology paired with radio frequency (RF) transmission (GNSS-RF). GNSS-RF transponders (Atlas PTs) receive positional information from GNSS satellites and relay this information to one another using radio frequency transmission. Atlas PT X is located at the geofence intersection point, while Atlas PTs A, B, and C are located at the triangle points A, B, and C, respectively. The manual faller carried a PT attached at the hip (Atlas PT F).

In this study, real-time geofence alert signals were evaluated in a random sample of 21 stands on the University of Idaho Experimental Forest (UIEF) (Fig 1A). Only stands ≥ 2.02 hectares (5 acres) in size were selected. Within each stand, the timing of geofence alerts was characterized for a manual faller entering a 100 m × 300 m rectangular geofence (Fig 1B). In addition to placing one stationary PT at the geofence intersection point (Atlas PT X in Fig 1B) to record data, a compass and 100-m fiberglass tape were used to place three other PTs 100 m from the virtual boundary intersection point at angles forming the vertices of an equilateral triangle (Atlas PTs A, B, and C at triangle points A, B, and C, respectively, in Fig 1B). The first of these stationary PTs (PT A) was placed at a randomly selected bearing from the intersection point (triangle point A). The bearing was sampled from the set of whole numbers between 1 and 360, with replacement. The remaining two stationary PTs (PTs B and C) were placed 120° and 240° clockwise, respectively, from this first PT (triangle points B and C, respectively). All stationary PTs were zip-tied to wooden stakes such that the bottoms of their antennas were 1 m above the ground surface. Each stationary PT was attached to a Windows 10 Dell Venue 8 Pro tablet running Raveon RavTrack software. Finally, maps for each stand were loaded onto the tablets using 1-m resolution National Agriculture Imagery Program (NAIP) images downloaded from The National Map website [52] and all tablets were synced with the National Institute of Standards and Technology (NIST) time server [53] each day.

The orientation of geofence crossing in each stand was also randomly selected from the sample of whole numbers between 1 and 360, with replacement. A rectangular geofence was established in each stand using a Suunto sight-through azimuth compass, fiberglass tape, and an Eos Arrow 100 GNSS unit (Eos Positioning Systems, Terrebonne, Quebec, Canada) with a specified accuracy of < 0.6 m [54]. One side of the geofence was centered at the geofence intersection point and was oriented perpendicular to the crossing direction. The geofence was 100 m wide at the crossing point and 300 m long.

In each stand, a manual faller carrying a PT attached at the belt crossed the geofence once by walking a 90-m route oriented perpendicular to the geofence (i.e., in the chosen geofence crossing direction), starting and ending 45 m from the intersection point. For consistency, the manual faller walked at a pace of 45 bpm, as dictated by a digital metronome. The route was established using a compass and 50-m fiberglass tape and was marked with pin flags. The observed time at which the faller crossed the geofence was recorded in the field using a custom script in R [55] running on a Windows 10 Dell Venue 8 Pro tablet synced with the NIST time server. The predicted intersection times were recorded by the tablets attached to each stationary PT (PTs A, B, C, and X). All PTs were set to collect and transmit their coordinates at a rate of once per second.

**Fig 2:**
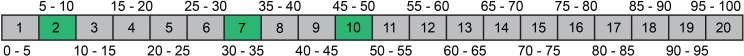
LOS path sections. Each LOS path was divided into 20 5-m sections and three sections were randomly selected for each LOS path. This figure shows the 20 sections and their locations along the LOS path. Sections highlighted in green represent the three randomly selected sections for which all vegetative obstructions were measured using the key in Table 1.

Within each stand, topographic, physical, and vegetative obstructions present along each LOS path between the geofence intersection point and the PTs located at triangle points A, B, and C were quantified during setup using a modification of the FIREMON line intercept method [56]. To mark the LOS path, a 100-m fiberglass tape was attached to two metal stakes. One stake was located at the intersection point and the second was located where the stationary PT would be placed during the experiment. The tape was secured to these two stakes and stretched taught 1 m above the ground to match the location of the stationary PT antenna height. This height was approximately equal to that of a PT when worn by a ground worker on a belt clip. The start of the tape was attached to the stake at the intersection point while the end was attached to the stake at the triangle point. For each LOS path, three 5-m sections were randomly selected from the segments shown in Fig 2 and all vegetative obstructions in these sections were classified using the key in Table 1. Only obstacles physically touching the fiberglass tape were quantified. To measure each obstacle, two meter sticks were used to hold the tape 1 m above the ground. The first meter stick was located 1 m before the start of the obstacle and the second meter stick was located 1 m past the end of the obstacle. Once the fiberglass tape was in position, the two locations at which the obstacle first and last contacted the tape were recorded to the nearest centimeter. Obstacles less than 5 cm in size (as measured along the LOS path) were not quantified. When gaps were present between nearby obstacles, the obstacles were treated as two separate obstacles only when the gap was greater than 25 cm. Obstructions less than 1 m tall were not quantified and trees that were less than 12.5-cm diameter at breast height (DBH) were classified as coniferous vegetation. To simplify analysis, vegetative obstructions within the three measured 5-m sections were summarized using Eq (1):

$$V_{i}=S_{i}+C_{i}+T_{i}+W_{i}+{SC}_{i}+{SW}_{i}+{CW}_{i}$$ (1)

Where *V*_*i*_ is the measured distance of all vegetative obstructions recorded for the three 5-m sections along the *i*th LOS path. *S*_*i*_, *C*_*i*_, *T*_*i*_, *W*_*i*_, *SC*_*i*_, *SW*_*i*_, and *CW*_*i*_ represent the distance of vegetative obstructions defined in Table 1 as measured in the three 5-m sections along the *i*th LOS path. Then, to account for the fact that only 15 m of each 100-m LOS path was measured, the total distance of vegetation along each path was calculated using Eq (2):

$${TV}_{i}=\frac{V_{i}}{0.15}$$ (2)

Where *TV*_*i*_ represents the total distance of vegetation along the entire *i*th LOS path and *V*_*i*_ is the measured distance of all vegetative obstructions recorded in the three 5-m sections along the *i*th LOS path (Eq (1)). Lastly, all boulders, streams, and forest roads were recorded as present or absent along each LOS path, regardless of their location on the path. These were recorded because of the effects they may have as terrain changes. However, because only one boulder was measured in the LOS paths, we removed it from analysis.

**Table 1:** Obstacle list and key.

Obstacle ID	Vegetative Obstruction
S	Deciduous shrub
C	Coniferous vegetation
T	Tree (stem)
W	Coarse woody debris (CWD)
SC	Deciduous shrub/coniferous vegetation
SW	Deciduous shrub/CWD
CW	Coniferous vegetation/CWD

**Fig 3:**
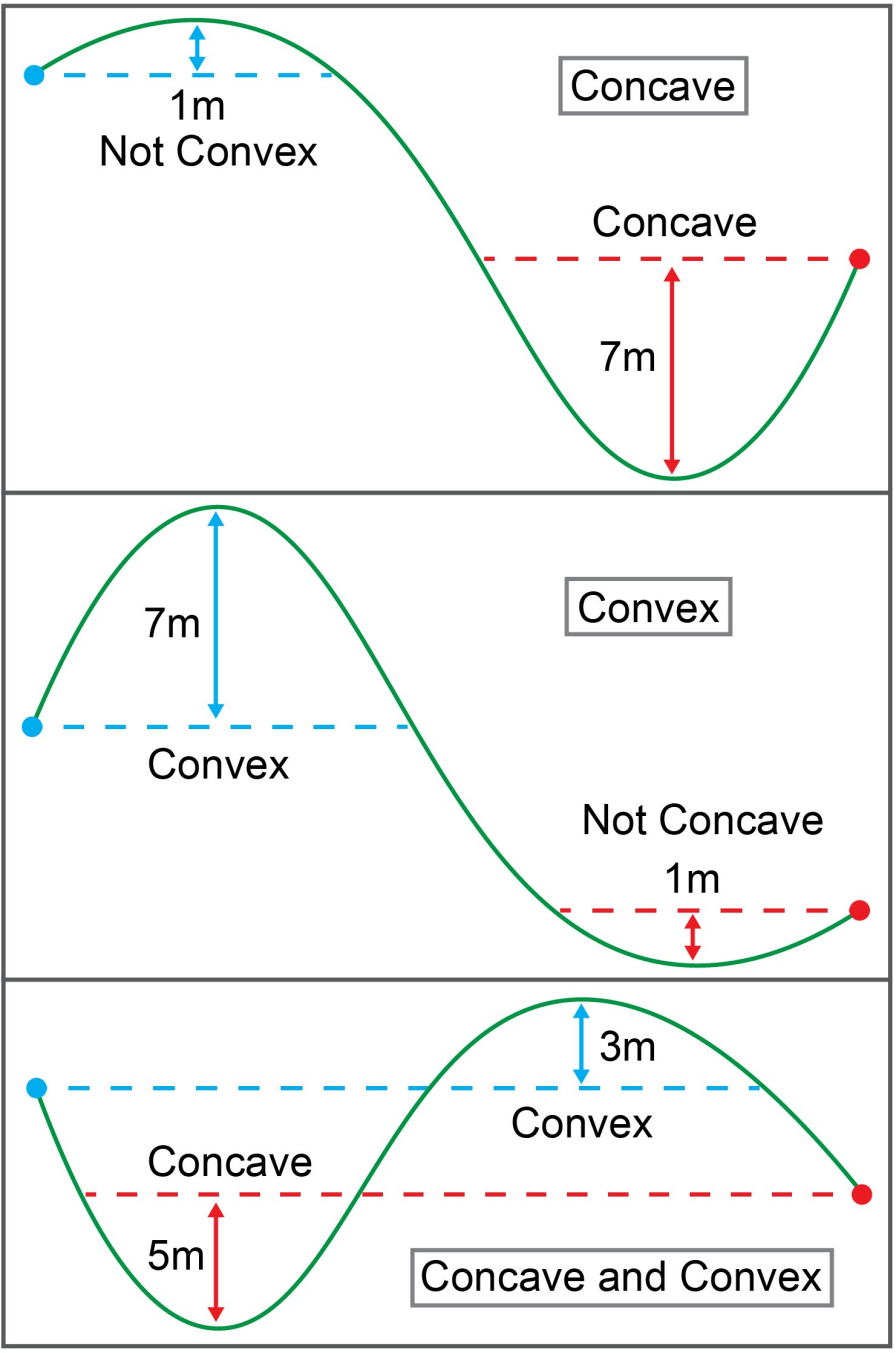
Illustration of slope classification as concave, convex, or both. Blue dots represent the higher of the two LOS endpoints while red dots represent the lower of the two LOS endpoints. Green lines represent the ground surface along the LOS path.

Once all obstructions were quantified and recorded, each LOS path was walked carrying a Garmin Alpha 100 GNSS-RF unit (Garmin, Olathe, Kansas, USA) to record the vertical elevation profile. Using the Garmin data, each LOS path was classified in terms of the presence or absence of concavity and convexity. A LOS path was concave if the minimum elevation along the path was at least 3 m below the lower of the two path endpoints. A LOS path was convex if the maximum elevation along the path was at least 3 m above the higher of the two path endpoints. The classification criteria for concavity and convexity is illustrated in Fig 3. The percent slope and aspect were also measured at the geofence intersection point. Aspect was measured as a continuous circular variable, but was reclassified as either N (316°– 45°), E (46°– 135°), S (136°– 225°), or W (226°– 315°). Trimble’s GNSS mission planning website [57] was used to determine the predicted PDOP values for each day during the experiment. Sampling was only conducted during times with predicted PDOP values less than 4.5 to ensure consistency.

To quantify forest stand characteristics, a 0.03-hectare fixed-area plot was established in each stand, centered at the intersection point. The DBH, total height, and height to the base of the live crown were quantified for all trees ≥ 12.5-cm DBH within the plot. Using these measurements, total basal area (TBA), trees per hectare (TPH), mean height (Ht), and quadratic mean diameter (QMD) were calculated for each stand and used as variables representing forest stand characteristics during analysis. Quadratic mean diameter is a commonly used metric in forestry that refers to the diameter of the tree of mean basal area (stem cross-sectional surface area), as measured at breast height (1.37 m). QMD is calculated as the square root of the squared stem diameters divided by the number of stems sampled, as defined in Eq (3):

$$QMD=\sqrt{\sum_{i=1}^{n}{D_{i}}^{2}/n}$$ (3)

Where *D*_*i*_ is the DBH of the *i*th tree and *n* is the number of trees sampled.

To quantify missed radio signals in each stand, the number of missed position updates transmitted from the faller’s PT (PT F) to the three PTs at the triangle points (PTs A, B, and C) was calculated for the 90-s interval centered on the observed geofence intersection time. Because all units were set to transmit their coordinates at 1-s intervals, 90 position updates would have been received in this time period in the absence of missed signals.

Stationary GNSS accuracy was summarized using RMSE, which is a common measure of GNSS positional error and represents the difference between the predicted and observed coordinates of a GNSS unit. In each stand, the predicted coordinates were obtained using the stationary PT located at the geofence crossing point (denoted as Atlas PT X in Fig 1B). These coordinates were collected once per second for 5 min prior to the time of geofence crossing. The observed (true) coordinates for each of these PTs were obtained using the Eos Arrow 100 GNSS unit described above. All coordinates were converted to the Universal Transverse Mercator (UTM) projection and then the RMSE for each stationary PT was calculated using Eq (4):

$${RMSE}_{i}=\sqrt{\sum_{i=1}^{n}{\left((x_{i}-{\hat{x}}_{ij}\right)}^{2}+{\left(y_{i}-{\hat{y}}_{ij}\right)}^{2})/n}$$ (4)

Where *RMSE*_*i*_ is the RMSE value in the *i*th stand, *x*_*i*_ is the observed easting value in the *i*th stand (i.e., the Arrow 100 easting coordinates), ${\hat{x}}_{ij}$ is the *j*th predicted easting value in the *i*th stand (i.e., the PT easting coordinates), *y*_*i*_ is the observed northing value in the *i*th stand (i.e., the Arrow 100 northing coordinates), *ŷ*_*ij*_ is the *j*th predicted northing value in the *i*th stand (i.e., the PT northing coordinates), and *n* is the total number of PT signals received in the *i*th stand.

The overall geofence intersection alert delay for each stand was derived by averaging the time-to-signal delay calculated at each of the three triangle points (A, B, and C) (Eq (5)):

$$D_{i}=\frac{\left(P_{ij}-O_{i})+(P_{ik}-O_{i})+(P_{il}-O_{i}\right)}{3}$$ (5)

Where *D*_*i*_ is the overall delay for the geofence intersection in the *i*th stand and *O*_*i*_ is the observed time at which the faller crossed the geofence in the *i*th stand, as recorded in the field. *P*_*ij*_ is the predicted intersection time in the *i*th stand at the *j*th triangle point, represented by the recorded alert at triangle point A. *P*_*ik*_ is the predicted intersection time in the *i*th stand at the *k*th triangle point, represented by the recorded alert at triangle point B. *P*_*il*_ is the predicted intersection time in the *i*th stand at the *l*th triangle point, represented by the recorded alert at triangle point C. Using this formula, positive delays indicate geofence crossing alerts that were triggered after the faller crossed the geofence and negative delays indicate geofence crossing alerts that occurred before the faller intersected the geofence. We used time-to-signal delay as an integrated measure of the accuracy of mobile GNSS units sharing their locations, which differs from RMSE calculated by Kaartinen et al. [11] using known reference points along a path.

### Analysis of missed radio signals

To test the null hypothesis that the probability of successful GNSS-RF signal propagation was not related to forest stand characteristics, topography, or obstructions in the line-of-sight, a binomial generalized linear mixed-effects model was used to evaluate relationships between the odds of missed signals as a function of vegetative LOS obstructions, topography, and forest stand characteristics. The model was fitted using the glmer function in the R lme4 package [58]. Variables included as fixed effects were the total distance of vegetation along each LOS path (*TV*_*i*_), TBA, TPH, Ht, QMD, slope, aspect, and the presence or absence of forest roads, streams, convex slopes, and concave slopes (Table 2). These variables were included because of their potential effect on the successful propagation of radio signals. To avoid errors with model convergence, the TPH variable was multiplied by a scalar of 0.01. The stand was used as a random effect to account for unobserved variation between stands. The response was the log odds of missed position updates along each LOS path during the 90-s interval centered around the observed geofence intersection time.

**Table 2:** Model parameters.

Variable	Category
*TV*_*i*_^a^	LOS obstruction
*TV*_*mean*_^b^	LOS obstruction
TBA	Forest stand characteristic
TPH	Forest stand characteristic
Ht	Forest stand characteristic
QMD	Forest stand characteristic
Slope	Topography
Aspect	Topography
Presence/absence of forest roads	Topography
Presence/absence of streams	Topography
Presence/absence of convex slopes	Topography
Presence/absence of concave slopes	Topography

^a^ *TV*_*i*_ was used only in the analysis of missed radio signals.

^b^ *TV*_*mean*_ was used only in the analysis of RMSE and geofence intersection alert delay.

### Analysis of RMSE

A linear mixed-effects model was also used to test the null hypothesis that neither forest stand characteristics, topography, nor physical obstructions affected GNSS accuracy. The model was fitted using the lmer function in the R lme4 package [58] using the day on which sampling occurred as a random effect to account for variation between days, as might occur due to changing satellite availability and geometry. Variables included as fixed effects in this model were total vegetation (*TV*_*mean*_), TBA, TPH, Ht, QMD, slope, aspect, and the presence or absence of forest roads, streams, convex slopes, and concave slopes (Table 2). *TV*_*mean*_ for each stand was calculated by averaging the total distance of vegetation (*TV*_*i*_) from the three LOS paths within each stand. Also, because variables recorded as either present or absent (forest roads, streams, convex slopes, and concave slopes) were quantified along each LOS path, these variables were also considered to be present in this stand-level analysis if they were present along any of the LOS paths. The response variable was the PT RMSE in each stand (*RMSE*_*i*_) calculated using Eq (4).

### Analysis of geofence intersection alert delay

To test the null hypothesis that neither forest stand characteristics, topography, nor physical obstructions affected the time-to-signal accuracy of geofence crossings, a linear mixed-effects model was used to quantify relationships between the magnitude of geofence intersection alert delay as a function of forest stand characteristics, topographic structure, and vegetative LOS obstructions. The model was fitted using the lmer function in the R lme4 package [58] using the day on which sampling occurred as a random effect to account for variation between days, as might occur due to changing satellite availability and geometry. Variables included as fixed effects were total vegetation (*TV*_*mean*_), TBA, TPH, Ht, QMD, slope, aspect, and the presence or absence of forest roads, streams, convex slopes, and concave slopes (Table 2). *TV*_*mean*_ for each stand was calculated by averaging the total distance of vegetation (*TV*_*i*_) from the three LOS paths within each stand. Also, because variables recorded as either present or absent (forest roads, streams, convex slopes, and concave slopes) were quantified along each LOS path, these variables were also considered to be present in this stand-level analysis if they were present along any of the LOS paths. The response variable was the overall intersection alert delay in each stand (*D*_*i*_) calculated using Eq (5).

### Model selection

For each of the three analyses, a full model was first fitted to the data using all fixed effect terms. These fixed effects were removed one at a time in order of highest *p*-values. The aictab function in the R AICcmodavg package [59] was used to compare all resulting models and the model with the lowest corrected Akaike Information Criterion (AICc) was selected. In terms of the geofence alert delay model, the first two models with the lowest AICc had fixed effect terms that were not significant (*p*-values > 0.05), in which case the model with the third lowest AICc was selected as the final model because all fixed effects had *p*-values ≤ 0.05. Inferences about all three final models were made using the lincon function in the R trtools package [60], which provides point estimates, standard errors, 95% confidence intervals, and *p*-values for each model term. In the case of the logistic regression model, the point estimates, standard errors, and confidence intervals were exponentiated to represent the effect of each variable on the odds of getting a missed signal.

## Results

### Analysis of missed radio signals

The proportion of missed radio signals ranged from 0/90 to 20/90, with a mean of 3.30/90. The mixed-effects logistic regression model with the lowest AICc had total distance of vegetation along the LOS path (*TV*_*i*_), TPH*0.01, convex, stream, road, and aspect as fixed effects (Table 3). All fixed effects affected the odds of missed signals (*p* ≤ 0.05). The odds of a missed signal decreased by a factor of 0.93 per unit increase in *TV*_*i*_ (*p* = 5.65 × 10^−7^), while the odds of a missed signal increased by a factor of 1.10 per unit increase in TPH*0.01 (*p* = 1.45 × 10^−3^). The odds of a missed signal were 1.61 times higher when a slope was convex vs. not convex (*p* = 3.58 × 10^−2^) and 2.00 times higher in the presence of roads (*p* = 2.34 × 10^−5^). In the presence of streams, the odds of a missed signal decreased by a factor of 0.66 (*p* = 3.16 × 10^−2^). The odds of a missed signal were 1.05 times higher on east, 2.17 times higher on north, and 2.92 times higher on west aspects (as compared to south aspects), although this effect was only significant on north (*p* = 9.99 × 10^−3^) and west (*p* = 1.65 × 10^−4^) aspects.

**Table 3:** Summary of mixed-effects logistic regression model using stand as a random effect and the odds of missed position updates as the response.

Model term	Estimate	SE	Lower CI	Upper CI	*t*-value	DF	*p*-value
(Intercept)	0.0187	0.2759	0.0109	0.0321	−14.4247	Inf	3.6198 × 10^−47^
*TV*_*i*_	0.9331	0.0138	0.9082	0.9588	−5.0028	Inf	5.6502 × 10^−07^
TPH*0.01	1.1048	0.0313	1.0391	1.1747	3.1837	Inf	1.4539 × 10^−03^
Convex^a^	1.6084	0.2264	1.0320	2.5067	2.0989	Inf	3.5823 × 10^−02^
Stream^a^	0.6552	0.1967	0.4455	0.9634	−2.1495	Inf	3.1597 × 10^−02^
Forest road^a^	2.0042	0.1644	1.4522	2.7659	4.2298	Inf	2.3395 × 10^−05^
Aspect (E)	1.0531	0.3147	0.5683	1.9515	0.1644	Inf	8.6941 × 10^−01^
Aspect (N)	2.1718	0.3011	1.2038	3.9182	2.5761	Inf	9.9911 × 10^−03^
Aspect (W)	2.9186	0.2843	1.6719	5.0950	3.7679	Inf	1.6464 × 10^−04^

Coefficient estimates, standard errors, and lower and upper bounds have been exponentiated to be on the odds scale.

^a^ Indicator variables represent the presence of each respective feature.

### Analysis of RMSE

**Fig 4:**
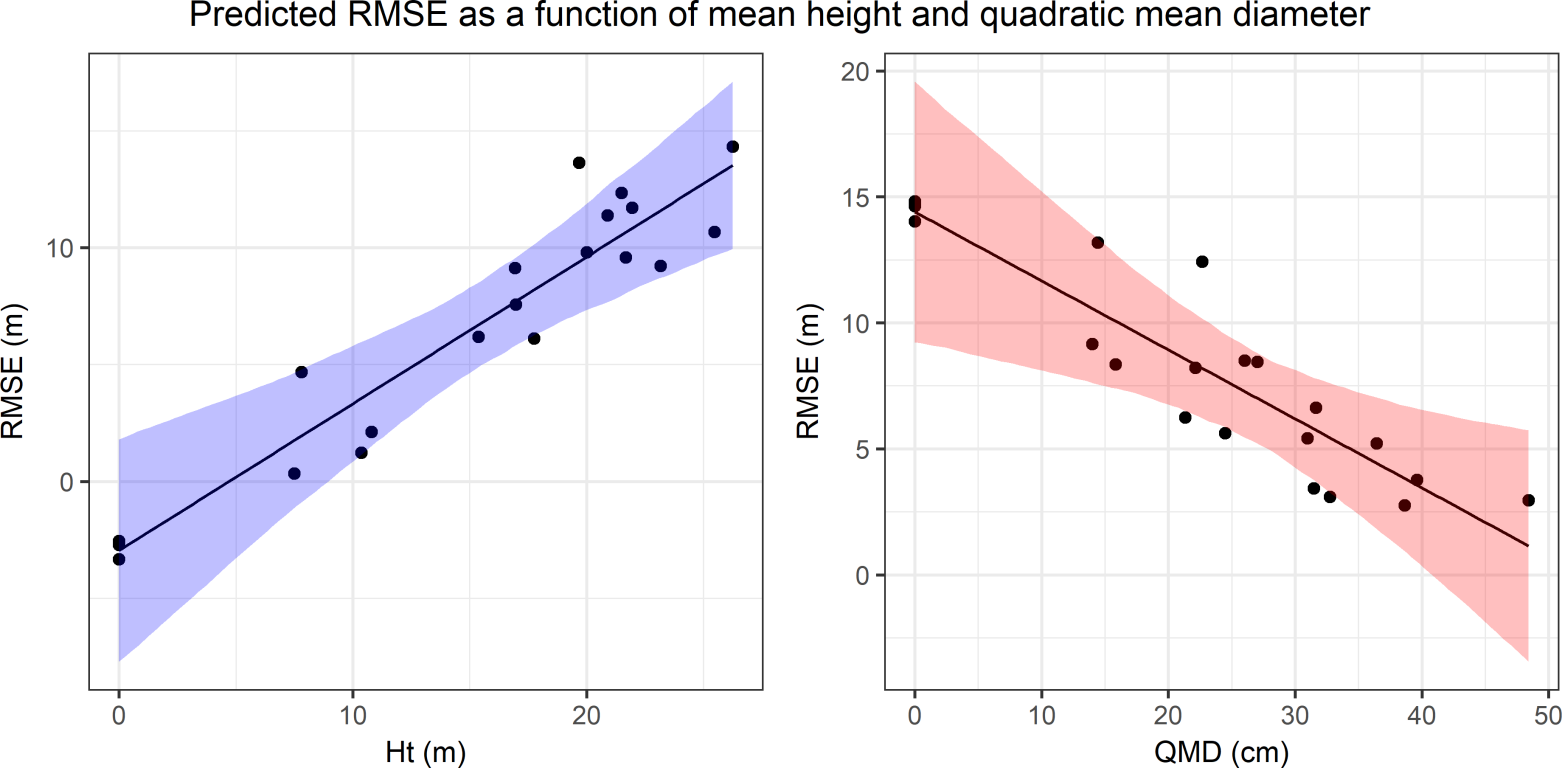
Mixed-effects model predictions for PT RMSE. Predicted RMSE as a function of the two model variables (Ht and QMD). Predictions for each variable were made using the mean of the other predictor. 95% confidence intervals computed using the bootstrap are shown as colored bands. Points on each plot represent partial residuals.

RMSE ranged from 1.81 m to 16.69 m, with a mean of 6.61 m. For the RMSE analysis, the mixed-effects model with the lowest AICc had Ht and QMD as fixed effects, both of which affected RMSE (*p* ≤ 0.05) (Table 4). The RMSE increased as Ht increased (*p* = 9.15 × 10^−6^) but varied indirectly with QMD (*p* = 3.02 × 10^−3^). Fig 4 illustrates the relationships between predicted RMSE as a function of the two explanatory variables included in the final mixed-effects model.

**Table 4:** Summary of mixed-effects linear regression model using day as a random effect and RMSE as the response.

Model term	Estimate	SE	Lower CI	Upper CI	*t*-value	DF	*p*-value
(Intercept)	3.7528	1.2790	1.2461	6.2595	2.9343	Inf	3.3435 × 10^−03^
Ht	0.6276	0.1415	0.3503	0.9048	4.4363	Inf	9.1526 × 10^−06^
QMD	−0.2737	0.0923	−0.4547	−0.0928	−2.9653	Inf	3.0243 × 10^−03^

### Analysis of geofence intersection alert delay

**Fig 5:**
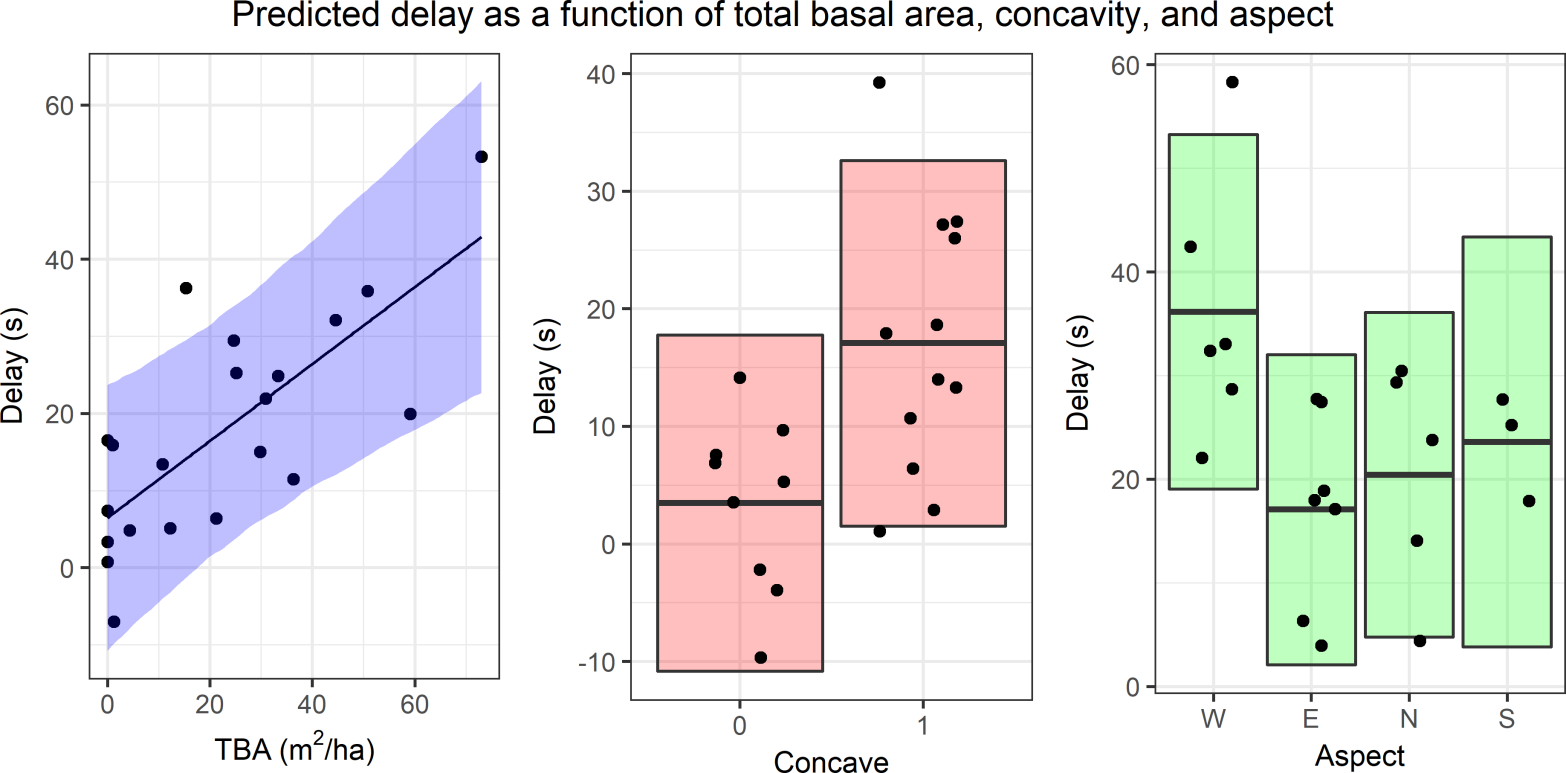
Mixed-effects model predictions for geofence intersection alert delay. Predicted delay as a function of the three model variables (TBA, concave, and aspect). Predictions for each variable were made using the mean of the other predictors. 95% confidence intervals computed using the bootstrap are shown as colored bands. Points on each plot represent partial residuals.

Geofence intersection alert delay ranged from −5.33 s to 66 s, with a mean of 18.62 s. The final mixed-effects model used to analyze the delay had TBA, concave, and aspect as fixed effects (Table 5). This model had the third lowest AICc, but was chosen because the two models with lower AICc values each had model terms that were not significant (*p* > 0.05). The geofence intersection alert delay increased as TBA increased (*p* = 6.49 × 10^−4^) and was also higher in the presence of concave slopes when compared to slopes that were not concave (*p* = 2.46 × 10^−2^). Finally, the delay was smaller on east, north, and south aspects (as compared to west aspects), although this effect was only significant on east aspects (*p* = 1.83 × 10^−2^). Fig 5 illustrates the relationships between the predicted alert delay as a function of the three explanatory variables included in the final mixed-effects model.

**Table 5:** Summary of mixed-effects linear regression model using day as a random effect and geofence intersection alert delay as the response.

Model term	Estimate	SE	Lower CI	Upper CI	*t*-value	DF	*p*-value
(Intercept)	11.9425	8.5931	−4.8998	28.7847	1.3898	Inf	1.6460 × 10^−01^
TBA	0.4988	0.1463	0.2121	0.7855	3.4104	Inf	6.4876 × 10^−04^
Concave^a^	13.5946	6.0494	1.7381	25.4512	2.2473	Inf	2.4622 × 10^−02^
Aspect (E)	−19.0832	8.0855	−34.9306	−3.2359	−2.3602	Inf	1.8267 × 10^−02^
Aspect (N)	−15.7249	9.4249	−34.1974	2.7475	−1.6684	Inf	9.5227 × 10^−02^
Aspect (S)	−12.5487	9.5119	−31.1916	6.0942	−1.3193	Inf	1.8708 × 10^−01^

^a^ Variable indicating the presence of concave slopes.

## Discussion

Analysis of missed radio signals indicated that forest stand characteristics, topography, and LOS obstructions affected the odds of missed signals. The odds of missed radio signals varied directly with stand density (TPH) and varied indirectly with LOS obstructions (*TV*_*i*_). Because previous work has shown that radio signal attenuation increases with greater vegetation depth and density, this result is somewhat counterintuitive [42–45]. However, the magnitude of these effects on the odds ratio was relatively small and may be a result of an interaction between stand density and vegetation in the LOS and/or external influences from wind or humidity [42,43,46–48]. Furthermore, topography influenced radio signal propagation, as convex slopes, aspect, and the presence of forest roads and streams affected the odds of missed signals. The mean number of missed signals during the observed 90-s interval was small, suggesting that relatively few position updates are missed at short distances (100 m).

Analysis of the PT RMSE indicated that only stand variables affected stationary GNSS accuracy. RMSE varied directly with Ht, but decreased with increasing QMD. Because characteristics associated with increasing forest stand density, such as canopy cover, are known to reduce GNSS accuracy, we expected that both predictors would have positive relationships with RMSE. Thus, this result is counterintuitive and may be an artefact of the data. A few sampled stands had relatively open canopies with large, mature trees at low density that may have affected the relationship between RMSE and QMD.

Both stand and topographic variables affected geofence intersection alert delay. Because of the way time delays were calculated, positive delays represent late alerts while negative delays represent early alerts. The alert delay varied directly with TBA and was higher in the presence of concave slopes. Aspect also affected alert delay, with delay being smaller on east slopes compared to west slopes. Taken together these results show that the time-to-signal accuracy of GNSS-RF geofence crossings is affected by both GNSS accuracy and radio signal propagation.

The relationships among the response and predictor variables were not strong for any of the three models. In the case of the missed radio signals, this could be due to the fact that the proportion of missed signals was generally low and the distance between radios fairly small (100 m). In terms of alert delay and RMSE, it is important to note that PTs receive coordinates from NAVSTAR GPS satellites only. Newer GNSS devices receive coordinates from multiple satellite constellations (i.e., from GPS, GLONASS, and BeiDou), which may improve accuracy and reliability in forested environments [61,62]. We collected data for a single geofence crossing in each stand, and RMSE measurements were collected for 5 min at each location. It is possible that clearer relationships would be evident if RMSE was determined using data collected over a longer period of time. Furthermore, future work should perform similar experiments using GNSS-RF transponders capable of processing multiple satellite constellations, as this may yield stronger patterns.

Our results suggest that GNSS-RF radio signal propagation is related to stand density, topography, and obstructions in the line-of-sight and that geofence alert timing is related to stand characteristics and topography. This indicates that the accuracy and successful sharing of GNSS coordinates may change depending on stand conditions and topography, both of which vary on active timber sales. Thus, real-time positioning based on consumer-grade GNSS-RF units may improve general communication and situational awareness on logging operations by allowing ground workers and equipment operators to view the relative positions of nearby workers and machines in real-time on mobile devices. However, high-resolution, mission-critical safety applications of this technology (e.g., geofencing) are not yet advisable under mature forest conditions. Future work should focus on the development of correction methods that account for the effects of forest stand characteristics on GNSS accuracy and geofence alert delay. Previous work has shown that adjustments should also be made for the angle and speed at which a tracked object approaches a geofence [36,37]. Such correction methods may improve reliability and enable a broader range of uses of GNSS-RF technology for safety applications in natural resources, especially if combined with GNSS-RF transponders that communicate with multiple satellite systems. Moreover, phone-based GNSS positioning that incorporates inertial navigation system (INS) capabilities has the potential for improving positioning accuracy. When paired with emerging Bluetooth-based augmentation technologies to create ad-hoc networks, these mobile-based solutions may be promising alternatives to dedicated GNSS-RF units for real-time positioning in remote environments.

## Supporting information

S1 Table Dataset used in analysis of missed radio signals. (XLSX)

S2 Table Dataset used in analysis of RMSE. (XLSX)

S3 Table Dataset used in analysis of geofence intersection alert delay. (XLSX)
